# Isolated renal hydatid cyst managed by laparoscopic transperitoneal nephrectomy

**DOI:** 10.4103/0970-1591.57925

**Published:** 2009

**Authors:** Kartik J. Shah, Arvind P. Ganpule, Mahesh R. Desai

**Affiliations:** Department of Urology, Muljibhai Patel Urological Hospital and Society for Research in Nephro-Urology, Dr. Virendra Desai Road, Nadiad, Gujarat – 387 001, India

**Keywords:** Hydatid cyst, laparoscopy, nephrectomy

## Abstract

Hydatid disease is a cyclozoonotic parasitic infestation caused by the cestode *Echinococcus granulosus*. Isolated renal involvement is extremely rare. A 45-year-old female, working as a farmer, presented with vague abdominal pain and hydatiduria. Ultrasonography of the abdomen revealed hydatid cyst arising from the right kidney. Computerized tomography scan of the abdomen confirmed the findings. Laparoscopic transperitoneal nephrectomy was performed. Isolated right renal hydatid cyst was removed *in toto*. Microscopic examination confirmed the diagnosis of hydatid cyst. Transperitoneal laparoscopic approach gives a better working space which helped us to remain outside Gerota's fascia and prevent subsequent cyst rupture.

## INTRODUCTION

Echinococcosis or hydatid disease is a cyclozoonotic parasitic infestation caused by the cestode *Echinococcus granulosus*. Human infestation is caused by larval form, and not the adult form, which is found in the small intestine of dog and other canine animals. Kidney involvement in echionococcosis is extremely rare, it is the third commonest organ involved after the liver and the lungs, constituting only 2-3% of all cases, even in areas where hydatid disease is endemic.[1] Isolated renal involvement is even rarer.

We present a case of a 45-year-old female, with isolated renal echinococcosis, a very rare presentation, and its management by laparoscopic transperitoneal nephrectomy

## CASE REPORT

A 45-year-old female patient presented with vague abdominal pain of 10-year duration, insidious in onset, nonprogressive and dull aching in type. She had a history of intermittent passage of small, white, grape-sized, balloon-like structures in the urine (hydatiduria) for last 10 years (twice a month).

**Figure 1:**
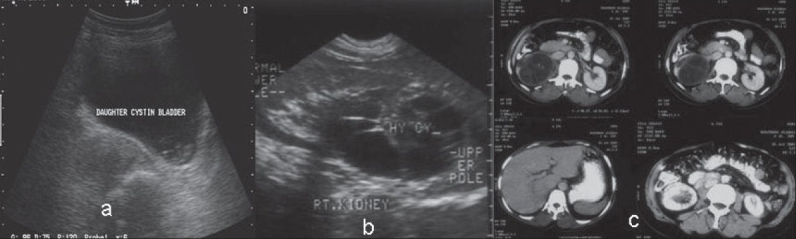
(a) Daughter cyst in bladder; (b) Hydatid cyst in right upper and midpole kidney; (c) Isolated hydatid cyst in right mid and lower pole kidney

Routine blood examination was normal. The X-rays of the abdomen and chest were normal. The ultrasonography (US) of the abdomen revealed a thin-walled multilocular cystic structure arising from upper and midpole extending to hilum of the right kidney measuring 7.4 × 6.7 × 6.5 cm in size. Lower calyx was dilated [Figure 1].

Computerized tomography (CT) scan [Figure 1b] of the abdomen confirmed the ultrasound findings. There were no similar lesions in other abdominal viscera. A diagnosis of isolated renal echinococcosis was made.

**Figure 2:**
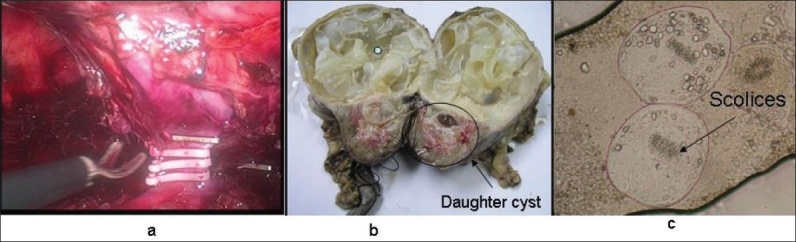
(a) Hilar dissection and division of vasculature; (b) Right nephrectomy specimen with daughter cyst in pelvis; (c) Microscopic appearance of scolices (H & E, 10×)

Laparoscopic transperitoneal nephrectomy was performed, under general anesthesia. Five ports (three – 11 mm, two – 5 mm) were placed. Approximately 75–80% of the right kidney was replaced by single hydatid cyst. Dense adhesions were present around the renal hilum. Renal vessels were secured with hem-o-lock clips. The specimen was retrieved in a specimen-retrieval bag and a thorough lavage was given with normal saline. The specimen was retrieved by extending the 11 mm working port. The postoperative recovery was uneventful [Figure 2a].

The histopathology revealed upper and middle pole kidney parenchyma being replaced by a unilocular cyst, measuring 6 cm in diameter and filled with daughter cyst. The outer wall was calcified. The cyst was communicating with the pelvicalyceal system. This confirmed the diagnosis of renal hydatid disease [Figure 2b and c].

## DISCUSSION

Common urology presentation of renal hydatidosis is of chronic dull flank or lower back discomfort from cystic pressure, it rarely presents as with hydatiduria, ureteropelvic junction obstruction,[2] and chronic renal failure.[3]

Ultrasonography, CT, and serologic investigations have facilitated the diagnosis of renal hydatid cysts. US is the most appropriate method for the differential diagnosis of a renal cystic tumor with a sensitivity of 95% for diagnosis. It is safe and inexpensive. CT should be reserved for equivocal cases. Percutaneous aspiration of the cyst and antihelminthic agents, such as mebendazole and albendazole, have also been used to treat hydatid disease, although these agents do not always sterilize cyst contents and have side effects. Danger of rupture or spillage of the highly antigenic contents precludes aspiration of hydatid cyst.[4]

Surgical options for renal hydatid cyst include total excision, partial nephrectomy, partial cystectomy, followed by capittonage. Renal sparing surgery of partial excision is possible in 75% of cases.[5]

In our case, real-time US revealed mobility of the kidney. Similarly, CT scan [Figure 2b] did not show perinephric stranding/adhesions. So, we decided to approach the kidney through a laparoscopic transperitoneal approach.

We believe from experience of our last 1000 laparoscopic cases dealt with transperitoneal approach that transperitoneal gives a better working space, which helped us to remain outside Gerota's fascia and prevent subsequent cyst rupture.

Although cases have been reported where renal hydatid cysts had been removed retroperitoneally. Our case is probably the first managed by laparoscopic transperitoneal route.

## CONCLUSION

Laparoscopic treatment is feasible, safe, and as effective as its open counterpart. This case is unique as isolated renal hydatid is rare and there are very few reports of laparoscopic management for this entity.[6]
